# Antibiotic Utilization Patterns and Appropriateness of Antibiotic Prescribing in an Internal Medicine Service of a Secondary Care Hospital: A Retrospective Drug Utilization Study

**DOI:** 10.3390/antibiotics15090931

**Published:** 2026-09-19

**Authors:** Ana Luisa Robles-Piedras, Leonardo Rosas-Villedas, Urias Bautista-Sánchez, José Roberto Medécigo-Hernández, Leonel Jeshua Vazquez-Ortiz, Elena Guadalupe Olvera-Hernández, Alejandro Chehue-Romero

**Affiliations:** 1 Academic Area of Pharmacy, Institute of Health Sciences, Autonomous University of the State of Hidalgo, San Agustín Tlaxiaca C.P. 42160, Hidalgo, Mexico; ro384326@uaeh.edu.mx (L.R.-V.); va476672@uaeh.edu.mx (L.J.V.-O.); olverae@uaeh.edu.mx (E.G.O.-H.); chehuea@uaeh.edu.mx (A.C.-R.); 2 General Hospital “Dra. Columba Rivera Osorio”, Instituto de Seguridad y Servicios Sociales de los Trabajadores del Estado, Mexico-Pachuca Highway Km. 86.5, Col. Instituto de Seguridad y Servicios Sociales de los Trabajadores del Estado, Pachuca de Soto C.P. 42083, Hidalgo, Mexico; jrmedecigo@hotmail.com

**Keywords:** antimicrobial stewardship, drug utilization study, internal medicine, appropriateness of antibiotic prescribing, inappropriate prescribing, secondary care hospital, Mexico

## Abstract

Background/Objectives: Antimicrobial resistance poses a severe global threat, particularly in low- and middle-income countries. Hospitalized patients in internal medicine wards are vulnerable to inappropriate antibiotic exposure. This study aimed to evaluate antibiotic utilization patterns, standardize volumetric consumption, and assess the appropriateness of antibiotic prescribing against national guidelines in the Internal Medicine service of a secondary-care hospital in Mexico. Methods: A retrospective descriptive drug-utilization study based on hospital records was conducted from July 2023 to December 2024. During this period, 1127 admissions to the Internal Medicine service were recorded; 413 eligible patients who received at least one systemic antimicrobial agent were included, accounting for 769 prescription events. Antimicrobial consumption was expressed as Defined Daily Doses (DDDs) per 100 admissions according to WHO ATC/DDD methodology. A focused prescribing-appropriateness assessment was performed for the five most frequently prescribed antimicrobial agents (*n* = 439 prescription events). Results: The cohort had a mean age of 61.4 ± 18.2 years, and 51.3% were aged ≥65 years. Cephalosporins (30.4%) and carbapenems (16.1%) were the most frequently prescribed pharmacological classes. Ceftriaxone had the highest standardized utilization density (49.69 DDDs/100 admissions) and an 80.6% prescribing-alignment rate. Meropenem followed with 44.72 DDDs/100 admissions despite substantially fewer prescription events. The largest proportions of prescription events classified as opportunities for optimization were observed for levofloxacin (48.5%), vancomycin (41.4%), and meropenem (36.1%). Conclusions: High overall guideline alignment (73%) coexisted with substantial use of broad-spectrum antimicrobials. Stewardship prioritization should integrate quantitative utilization with qualitative prescribing assessment rather than interpret total DDD burden as avoidable exposure. These findings provide a baseline for targeted antimicrobial stewardship and clinical-pharmacy interventions in a resource-constrained secondary-care setting.

## 1. Introduction

The global emergence and rapid dissemination of antimicrobial resistance (AMR) represent one of the most formidable threats to modern medicine, compromising therapeutic efficacy, increasing morbidity, and escalating healthcare-related costs [1]. The Global Burden of Disease 2021 antimicrobial resistance analysis estimated that bacterial AMR was directly attributable to approximately 1.14 million deaths and associated with approximately 4.71 million deaths worldwide in 2021 [2]. Under the reference forecast, annual deaths attributable to bacterial AMR are projected to reach approximately 1.91 million by 2050, with approximately 8.22 million deaths associated with AMR [3]. These contemporary estimates underscore the need for coordinated interventions to optimize antimicrobial use, strengthen infection prevention, and improve access to timely diagnostics and effective therapies. While resistance is an inevitable evolutionary phenomenon driven by bacterial adaptation and horizontal gene transfer, its acceleration is primarily fueled by complex anthropogenic factors, most notably antimicrobial overuse and prescribing challenges within community and hospital ecosystems [4]. In acute care hospital settings, a substantial proportion of inpatient antibiotic prescriptions present opportunities for therapeutic optimization in molecule selection, dosing, administration route, or duration, representing an important target for institutional quality improvement [5].

To mitigate this impending therapeutic crisis, the World Health Organization (WHO), along with international infectious disease societies, has strongly advocated for the universal institutionalization of Antimicrobial Stewardship Programs (ASPs), nationally designated as Programas de Optimización de Uso de Antimicrobianos (PROA) in Spanish-speaking healthcare networks [6,7]. ASPs are designed as structured, multidisciplinary, and data-driven institutional frameworks that aim to optimize clinical outcomes, minimize drug-related toxicities, and suppress the selective pressure for resistance by supporting the clinical staff in ensuring the “five rights” of antimicrobial therapy: the right drug, for the right patient, at the right dose, via the right route, and for the right duration [8]. Within these programs, the integration of the clinical pharmacist has proved to be a fundamental cornerstone for success, facilitating real-time collaborative interventions such as pharmacokinetic/pharmacodynamic (PK/PD) optimization, renal dose adjustments, intravenous-to-oral sequential therapy, and systematic post-prescription reviews [9]. Nevertheless, the operationalization of robust ASPs in LMICs, particularly in Latin America, faces profound structural barriers, including resource limitations, restricted access to rapid microbiological diagnostics, and a historical reliance on empirical prescribing driven by the acute nature of infectious diseases [10,11].

Within the complex ecosystem of a secondary care hospital, the Internal Medicine service represents a critical and high-yield focal point for stewardship implementation. This ward routinely manages a highly heterogeneous inpatient population characterized by advanced chronological age, physiological frailty, and multiple chronic comorbidities, such as type 2 diabetes mellitus, chronic kidney disease, congestive heart failure, and severe hypertension [12,13]. These clinical attributes profoundly alter the baseline vulnerability of these patients, predisposing them to healthcare-associated infections (HAIs), recurrent sepsis, and adverse drug events. Furthermore, the intersections of immunosenescence and altered pharmacokinetics, such as diminished glomerular filtration rates, fluctuating volumes of distribution, and erratic protein binding, render the selection and dosing of antibiotics in these patients exceptionally challenging [14]. Because older adults frequently exhibit atypical clinical presentations of infections, physicians in internal medicine wards often initiate broad-spectrum, empirical antimicrobial regimens defensively to protect patient safety during critical windows of care, which frequently extend beyond traditional clinical guidelines in the absence of timely diagnostic de-escalation tools [15].

From a pharmacoepidemiological perspective, monitoring specific antimicrobial classes within these wards is paramount due to their varying ecological and clinical footprints. Fluoroquinolones, particularly levofloxacin, are frequently utilized in internal medicine due to their excellent bioavailability, convenient once-daily dosing, and broad-spectrum coverage against respiratory and urinary pathogens [16]. However, their extensive use is strongly associated with the selection of multidrug-resistant (MDR) Gram-negative bacilli and potential systemic toxicities, prompting safety updates from global regulatory bodies regarding aortic aneurysms, tendonitis, and dysglycemia in vulnerable populations [17]. Similarly, lincosamides like clindamycin are prescribed empirically for suspected anaerobic or skin and soft tissue infections, yet they represent a potent trigger for Clostridioides difficile infections (CDI) in hospitalized older adults [18]. In contrast, high-alert, reserve antimicrobials such as carbapenems (meropenem, imipenem) and glycopeptides (vancomycin) require careful institutional monitoring policies, as their utilization patterns directly impact the emergence of carbapenem-resistant Enterobacteriaceae (CRE) and vancomycin-resistant Enterococci (VRE), frequently leaving clinicians with highly restricted therapeutic alternatives [19,20].

In Mexico, federal strategic efforts have been codified to regulate antimicrobial prescribing, including the landmark 2010 decree mandating prescription-only community sales and the 2018 establishment of the National Strategy Against Antimicrobial Resistance [21,22]. Despite these legislative frameworks, a significant operational gap remains between centralized policy and the daily clinical reality of secondary care public hospitals, which frequently navigate structural budget constraints and lack formalized, multi-disciplinary ASP committees to support medical decision-making [23]. Quantitative and qualitative Drug Utilization Studies (DUSs) serve as indispensable diagnostic instruments to map local prescribing habits, quantify the precise consumption of critical therapeutic classes, and measure adherence to national clinical guidelines (such as those from CENETEC and IMSS) from a perspective of continuous quality improvement [24]. Consequently, generating granular, baseline data regarding appropriateness of antibiotic prescribing is an urgent prerequisite for designing targeted, institution-specific collaborative protocols. To address this knowledge gap, the objective of this study was to evaluate the patterns of antibiotic utilization, characterize the clinical-demographic profile of the treated population, and determine the rates of appropriateness of antibiotic prescribing against established national guidelines within the Internal Medicine service of a public secondary care hospital in Mexico, thereby providing a baseline to justify the formal integration of clinical pharmacy as a strategic asset for patient safety and medical staff support.

## 2. Results

The findings of this drug utilization study are presented below, encompassing the demographic characteristics of the evaluated patient cohort, the quantitative patterns of antibiotic consumption, and the qualitative assessment of the appropriateness of antibiotic prescribing within the Internal Medicine ward.

### 2.1. Demographic and Clinical Characterization of the Study Population

During the study period from July 2023 to December 2024, 1127 admissions to the Internal Medicine service were recorded. A census-based screening identified 413 unique hospitalized patients who met the eligibility criteria of being older than 18 years, receiving at least one systemic antimicrobial agent during hospitalization, and having sufficient clinical and pharmacological information for evaluation. These patients accounted for 769 individual antimicrobial prescription events.

As summarized in Table 1, the demographic distribution of the cohort showed a slight predominance of female patients (*n* = 221, 53.51%) compared to male patients (*n* = 192, 46.49%). The study population exhibited a distinctly geriatric profile, with a mean age of 61.4 ± 18.2 years, and patients aged 65 or older constituted the absolute majority of the sample (*n* = 212, 51.33%). The mean length of hospital stay (LOS) for the entire cohort was established at 7.8 ± 4.3 days, reflecting the high clinical complexity and chronicity typically managed within secondary-care internal medicine units.

### 2.2. Antibiotic Consumption Patterns and DDD Metrics

Third-generation cephalosporins constituted the most frequently prescribed antimicrobial class. Ceftriaxone was identified as the single most prescribed active pharmaceutical ingredient (*n* = 160 prescriptions), followed by intravenous meropenem (*n* = 72) and vancomycin (*n* = 70). Significant discrepancies were observed when comparing the total calculated grams consumed in the hospital sample against the reference Defined Daily Dose (DDD) values established by the WHO/ATC system, as detailed in Table 2.

Ceftriaxone exhibited the highest standardized utilization density within the Internal Medicine service, reaching 49.69 DDDs/100 admissions. Meropenem followed closely at 44.72 DDDs/100 admissions despite having less than half the number of prescription events observed for ceftriaxone. This finding illustrates the value of complementing prescription-event frequency with standardized utilization metrics when characterizing antimicrobial use.

Piperacillin/tazobactam accounted for the largest absolute active-substance mass processed during the 17-month period (5796 g), corresponding to 36.73 DDDs/100 admissions. Vancomycin and levofloxacin showed lower standardized utilization densities of 21.74 and 19.96 DDDs/100 admissions, respectively.

### 2.3. Antimicrobial Utilization Patterns by Pharmacological Families

**Figure 1:**
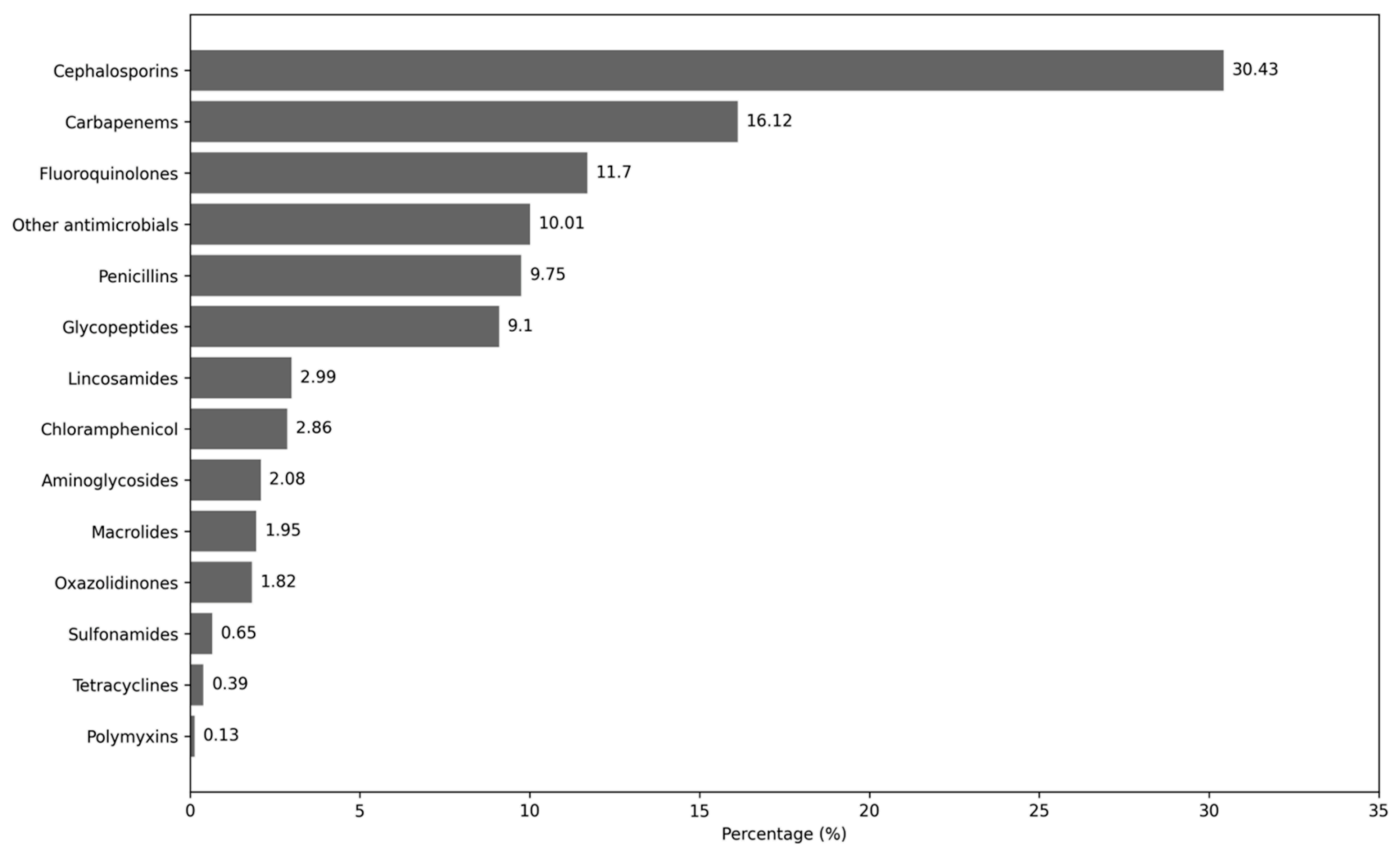
Distribution of antimicrobial prescription events by pharmacological class (%) within the Internal Medicine service (*n* = 769 prescription events).

The pharmacoepidemiological screening identified 14 antimicrobial pharmacological classes within the hospital service. Prescription events were concentrated mainly among cephalosporins (*n* = 234, 30.43%), carbapenems (*n* = 124, 16.12%), fluoroquinolones (*n* = 90, 11.70%), and penicillins (*n* = 75, 9.75%). Glycopeptides accounted for 70 prescription events (9.10%), while the remaining classes had lower frequencies. The complete percentage distribution of these 14 pharmacological classes is presented in Figure 1.

### 2.4. Distribution of Primary Antimicrobials by Clinical Pathology

To contextualize clinical decision-making within the local epidemiological environment, the five most frequently prescribed individual antimicrobial agents were cross-tabulated against the primary infectious pathologies documented in this cohort. As detailed in Table 3, prescription events among these five agents were most frequently associated with pneumonia (*n* = 118) and urinary tract infections (UTIs) (*n* = 87).

### 2.5. Evaluation of Antibiotic Prescribing Appropriateness and Optimization Indicators

**Figure 2:**
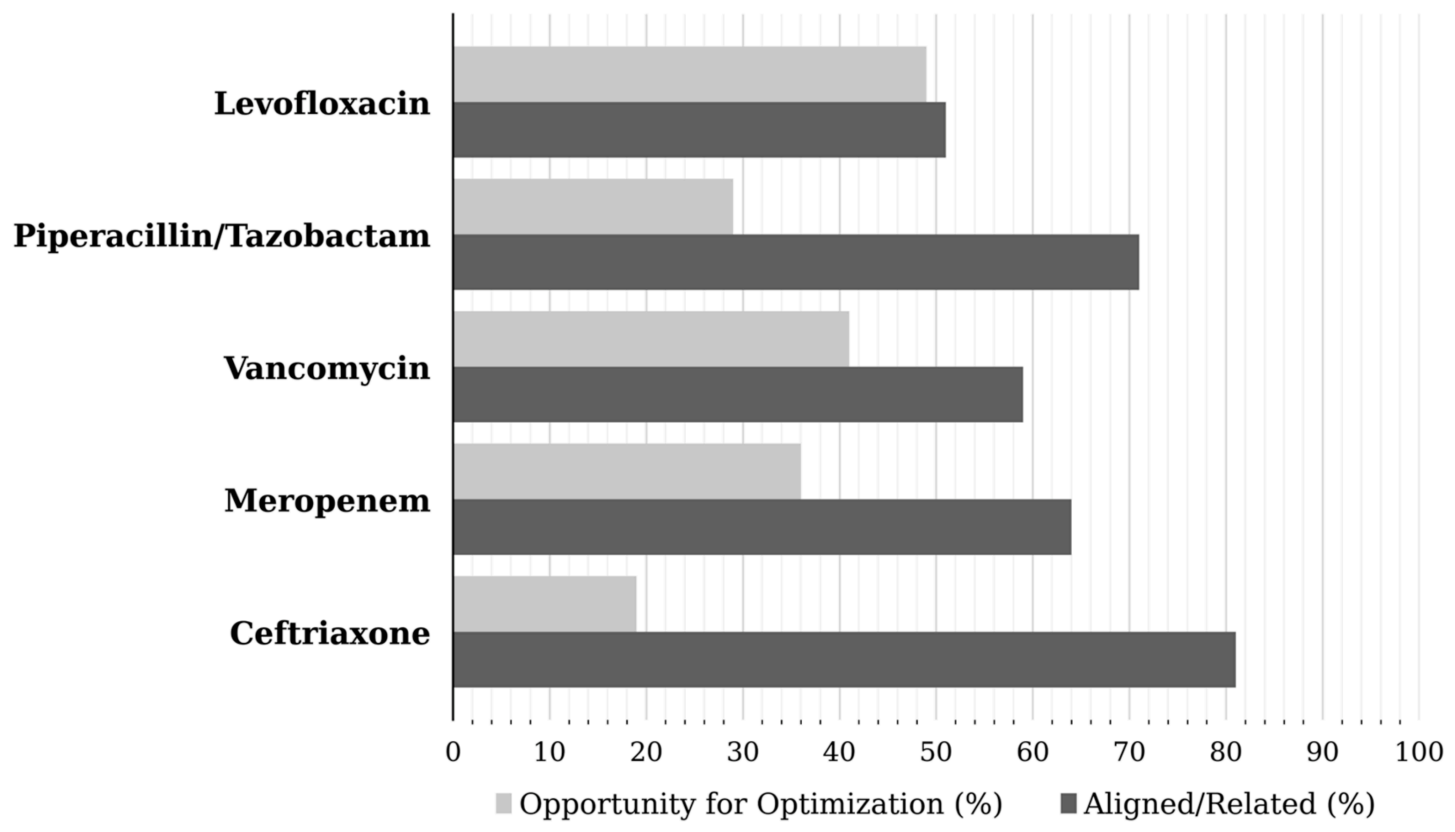
Proportional breakdown of antibiotic prescribing appropriateness and opportunities for therapeutic optimization among the five most frequently prescribed antimicrobial agents (*n* = 439 prescription events).

When the 769 total prescription events were contrasted against national clinical practice guidelines and institutional protocols, 73% were categorized as “Aligned/Related” to the documented clinical diagnosis. To identify specific institutional targets for intervention, a focused assessment was performed for the five most frequently prescribed antimicrobial agents (*n* = 439 prescription events). As illustrated in Figure 2, Ceftriaxone demonstrated the highest alignment rate, with 80.62% of its 160 prescriptions classified as “Aligned/Related,” followed by piperacillin/tazobactam (*n* = 69) with 71.01% alignment. Meropenem (*n* = 72) and vancomycin (*n* = 70) showed opportunities for optimization in 36.1% and 41.4% of prescription events, respectively. The largest proportion was observed for levofloxacin (*n* = 68), for which 48.5% of prescription events were classified as opportunities for optimization. These findings identify specific targets for structured post-prescription review, diagnostic reassessment, dose optimization, and timely de-escalation.

### 2.6. Identification of Critical Priority Areas for Institutional Intervention

The prioritization framework was revised to distinguish overall antimicrobial utilization burden from potentially modifiable prescribing. Quantitative utilization and qualitative prescribing-appropriateness indicators were therefore interpreted together rather than assuming that total antimicrobial exposure represented avoidable exposure.

Ceftriaxone remained the antimicrobial with the highest utilization density (49.69 DDDs/100 admissions); however, its high prescribing alignment rate (80.62%) indicates that total ceftriaxone exposure should not be interpreted as largely avoidable. In contrast, levofloxacin, vancomycin, and meropenem showed higher proportions of prescription events classified as opportunities for optimization (48.5%, 41.4%, and 36.1%, respectively). These agents therefore represent higher-priority targets for focused stewardship review, particularly regarding indication, spectrum selection, dose optimization, and timely de-escalation. Because the dataset did not permit patient-level attribution of DDDs to each appropriateness category, potentially avoidable DDDs were not estimated directly.

## 3. Discussion

This retrospective drug-utilization study provides a multidimensional baseline of antimicrobial prescribing in the Internal Medicine service of a Mexican secondary-care public hospital. By integrating prescription-event frequencies, standardized utilization densities (DDDs/100 admissions), and qualitative prescribing-appropriateness indicators, the analysis shows that relatively high overall guideline alignment can coexist with substantial use of broad-spectrum agents. This type of combined assessment is useful for moving from purely empirical prescribing toward targeted Antimicrobial Stewardship Programs (ASPs/PROA) in resource-constrained settings [25]. The cohort had a mean age of 61.4 ± 18.2 years, and 51.33% of included patients were aged 65 years or older, with a mean length of stay of 7.8 ± 4.3 days. In older adults, immunosenescence, multimorbidity, and age-related pharmacokinetic changes can increase both the risk of therapeutic failure and adverse drug events [26], complicating antimicrobial selection and dosing when timely microbiological information is unavailable.

Cephalosporins (30.43% of prescription events) and carbapenems (16.12%) were the most frequently represented classes. After correction of the denominator to all 1127 Internal Medicine admissions, ceftriaxone showed the highest standardized utilization density (49.69 DDDs/100 admissions), followed by meropenem (44.72 DDDs/100 admissions). The relatively high use of ceftriaxone is consistent with its frequent use in hospital practice and with reports describing prominent third-generation cephalosporin use in Mexican institutions [27,28]. Extensive cephalosporin exposure remains relevant from a stewardship perspective because of its potential ecological impact, including selection pressure for extended-spectrum beta-lactamase (ESBL)-producing Enterobacteriaceae [29,30]. Meropenem had substantially fewer prescription events than ceftriaxone but a standardized utilization density close to that of ceftriaxone, illustrating why prescription counts alone may underestimate the utilization burden of reserve agents. Recent Mexican point-prevalence studies provide important contemporary context. Zumaya-Estrada et al. reported high antibiotic use in secondary-care hospitals, with prescribing predominantly empirical and parenteral [31]. In a tertiary-care Mexican hospital, 47.5% of hospitalized patients were receiving antibiotics; 92.4% of prescriptions were empirical and 95.9% parenteral, with ceftriaxone the most frequently prescribed antimicrobial (18.9%) and meropenem also among the leading agents (8.2%) [32]. These national data are consistent with the prominent role of broad-spectrum empirical therapy observed in our service and reinforce the need for structured reassessment and de-escalation. These findings also highlight the importance of distinguishing total utilization from potentially modifiable prescribing. Ceftriaxone had the highest utilization density but also the highest alignment rate (80.62%); therefore, its total DDD burden should not be interpreted as avoidable exposure. By contrast, levofloxacin, vancomycin, and meropenem showed larger proportions of prescription events classified as opportunities for optimization (48.5%, 41.4%, and 36.1%, respectively). Stewardship prioritization should therefore integrate quantitative utilization metrics with qualitative prescribing assessment rather than rely on DDD burden alone. Levofloxacin remains an important target because unnecessary or prolonged fluoroquinolone exposure can increase ecological pressure and is associated with clinically important adverse effects, particularly in older adults [33,34,35]. Vancomycin prescriptions showed an optimization opportunity in 41.4% of evaluated events. Current consensus recommendations for serious methicillin-resistant Staphylococcus aureus (MRSA) infections favor area-under-the-curve (AUC)-guided therapeutic drug monitoring rather than trough-only monitoring, because AUC-guided dosing more directly balances efficacy and nephrotoxicity risk [36,37]. In this setting, the absence of structured AUC-based monitoring and individualized dose adjustment represents a relevant opportunity for antimicrobial stewardship and patient-safety intervention. The observed divergence from reference recommendations should not be interpreted as systemic prescriber failure. Secondary-care clinicians frequently work with service saturation, limited diagnostic resources, and delayed microbiological results, conditions that favor defensive broad-spectrum empirical therapy. Diagnostic uncertainty and delayed reassessment can contribute to prolonged broad-spectrum coverage in low- and middle-income settings [38].

Once a patient achieves clinical stabilization, the absence of an institutionalized team for systematic post-prescription review may contribute to unnecessary continuation of broad-spectrum or reserve agents. This operational gap supports the integration of the clinical pharmacist as a collaborative member of the multidisciplinary team. Evidence from Latin American settings indicates that pharmacist-driven antimicrobial stewardship can improve antibiotic prescribing through real-time therapeutic oversight and structured post-prescription review [39]. The pharmacist should therefore function as a clinical partner supporting medical decision-making rather than as a punitive administrative auditor.

Participation of the clinical pharmacist in daily rounds can support model-informed precision dosing (MIPD), renal dose adjustment, intravenous-to-oral switching, and timely diagnostic de-escalation. The study nevertheless has important limitations. It was conducted in a single secondary-care public hospital, which limits generalizability. Its retrospective design depends on the completeness and accuracy of medical records and therefore remains susceptible to information and misclassification bias. In addition, DDDs/100 admissions are an aggregate utilization measure and do not represent exact patient-level exposure, particularly when individualized dose adjustments are required. Records lacking diagnosis, antimicrobial agent, dose, route, or treatment-duration information were excluded to reduce misclassification. A major strength is the integration of prescription frequencies, standardized utilization metrics, and qualitative prescribing-appropriateness assessment, which provides a practical baseline for designing institution-specific stewardship interventions [40,41].

## 4. Materials and Methods

### 4.1. Study Design and Population

A retrospective descriptive drug-utilization study based on hospital records was conducted in the Internal Medicine service of a secondary-care teaching hospital in Hidalgo, Mexico, covering the consecutive 17-month period from July 2023 to December 2024. During this period, 1127 admissions to the service were recorded. Patients were eligible if they were older than 18 years, were hospitalized in the Internal Medicine service, and received at least one systemic antimicrobial agent during hospitalization. Medical records were excluded when clinical or pharmacological information was incomplete or insufficient to evaluate antimicrobial prescribing, including missing information on diagnosis, antimicrobial agent, dose, route of administration, or treatment duration. All eligible records were included using a census-based approach; no sampling procedure was applied. The final cohort comprised 413 unique patients and 769 antimicrobial prescription events.

### 4.2. Data Source and Extraction Process

Data were systematically extracted from institutional clinical records and pharmacy dispensing logs using a structured data-collection form developed specifically for this study. The form did not undergo formal validation. It captured: (a) demographic variables (biological sex and age); (b) clinical variables (documented admission and secondary diagnoses and primary infectious pathology); and (c) pharmacological variables (specific antimicrobial agent, pharmacological class, daily dose administered within 24 h, route of administration, and treatment duration in days).

### 4.3. Ethical Considerations and Institutional Protection

Institutional authorization to conduct the study and access the required hospital records was granted in writing by the Director of the participating hospital (Authorization No. FC/010724, dated July 2023). All patient-specific data were anonymized prior to analysis. The study involved retrospective analysis of secondary clinical and administrative data without direct patient contact or intervention. The identity of the participating institution was protected in the manuscript in accordance with institutional confidentiality and data-use restrictions.

### 4.4. Metric Calculations: Appropriateness and Consumption (DDD)

The appropriateness of antibiotic prescribing was assessed by contrasting the documented clinical diagnosis and antimicrobial regimen against national clinical practice guidelines (CENETEC and IMSS) and the institutional formulary. Prescription events were categorized as “Aligned/Related” when antimicrobial selection was consistent with the applicable reference recommendation, or as an “Opportunity for Therapeutic Optimization” when deviations in empirical coverage or spectrum selection were identified. The analysis was designed as a prescribing-quality screening and did not equate total antimicrobial utilization with avoidable exposure.

Antimicrobial consumption was standardized according to the World Health Organization (WHO) Anatomical Therapeutic Chemical/Defined Daily Dose (ATC/DDD) methodology using the 2026 ATC/DDD Index. Total DDDs were calculated by dividing the total amount of active substance consumed by the corresponding WHO-assigned DDD. For the five principal agents, the ATC codes were J01DD04 for ceftriaxone, J01DH02 for meropenem, J01CR05 for piperacillin/beta-lactamase inhibitor, J01XA01 for vancomycin, and J01MA12 for levofloxacin. Consumption density was expressed as DDDs/100 admissions, using all Internal Medicine admissions recorded during the study period (*n* = 1127) as the denominator.

Consumption density was calculated as follows: DDDs/100 admissions = (Total DDDs/1127 Internal Medicine admissions) × 100.

(1) $$\mathrm{DDD}\;\mathrm{per}\;100\;\mathrm{Admissions}=\left(\frac{\mathrm{Total}\;\mathrm{Grams}\;\mathrm{Consumed}/\mathrm{WHO}\;\mathrm{Assigned}\;\mathrm{DDD}}{\mathrm{Total}\;\mathrm{Admissions}\;\left(N\right)}\right)\times 100$$

### 4.5. Statistical Analysis

Categorical variables, including biological sex, antimicrobial pharmacological class, and prescribing-appropriateness classifications (Aligned/Related vs. Opportunity for Therapeutic Optimization), were summarized as absolute frequencies and percentages (*n*, %). Continuous variables, including age and treatment duration, were assessed descriptively and expressed as means with standard deviations ($\bar{X}$ ± SD). Data cleaning, database consolidation, calculation of active-substance totals, DDDs, and DDDs/100 admissions, and graphical/cross-tabulation analyses were performed using Microsoft Excel^®^ (Redmond, WA, USA). No comparative inferential tests were applied because the study was descriptive. Potential sources of bias included incomplete clinical documentation, retrospective data capture, and possible misclassification of prescribing appropriateness. To reduce information bias, data extraction used a predefined structured form, records missing essential variables were excluded, and prescribing assessment was performed against predefined national and institutional reference standards.

## 5. Conclusions

In conclusion, this retrospective drug-utilization study establishes a multidimensional baseline of antimicrobial use and appropriateness of antibiotic prescribing within the Internal Medicine service of a Mexican secondary-care public hospital. Overall guideline alignment was 73%, while ceftriaxone and meropenem showed the highest standardized utilization densities at 49.69 and 44.72 DDDs/100 admissions, respectively. The focused prescribing assessment identified the largest opportunities for optimization for levofloxacin (48.5%), vancomycin (41.4%), and meropenem (36.1%).

These findings demonstrate that total antimicrobial utilization and potentially modifiable prescribing are related but distinct stewardship dimensions. High utilization alone should not be interpreted as avoidable exposure. Combining standardized utilization metrics with prescribing-appropriateness assessment provides a more defensible basis for prioritizing interventions, particularly post-prescription review, spectrum optimization, individualized dosing, and diagnostic de-escalation.

The results support formal integration of the clinical pharmacist as a collaborative member of the multidisciplinary team. Clinical-pharmacy participation in daily rounds, renal dose adjustment, AUC-guided vancomycin monitoring when clinically indicated, intravenous-to-oral switching, and timely de-escalation may help reduce medication-related harm and support antimicrobial stewardship without compromising patient safety or clinical outcomes.

## Figures and Tables

**Table 1:** Demographic and clinical baseline characteristics of the hospitalized patient population (*N* = 413).

Baseline Characteristic	Value for Patient Cohort (*N* = 413)	Percentage (%)
Biological Sex		
Female	221	53.51
Male	192	46.49
Age Distribution		
Mean± SD (Years)	61.4 ± 18.2	-
Geriatric Population (≥65 years old)	212	51.33
Adult Population (<65 years old)	201	48.67
Length of Hospital Stay		
Mean ± SD (Days)	7.8 ± 4.3	-

**Table 2:** Antimicrobial consumption patterns expressed as Defined Daily Doses (DDDs) per 100 admissions within the Internal Medicine service.

Antimicrobial Drug	Route of Admin.	WHO Assigned DDD (g)	Total Grams Consumed	Total DDDs	DDDs/100 Admissions
Ceftriaxone	IV	2.0	1120	560	49.69
Meropenem	IV	3.0	1512	504	44.72
Piperacillin/Tazobactam	IV	14.0 *	5796	414	36.73
Vancomycin	IV	2.0	490	245	21.74
Levofloxacin	IV/PO	0.5	112.5	225	19.96

* Note: WHO ATC/DDD Index 2026 values were used. ATC codes: ceftriaxone, J01DD04; meropenem, J01DH02; piperacillin/beta-lactamase inhibitor, J01CR05; vancomycin, J01XA01; levofloxacin, J01MA12. For piperacillin/tazobactam, the WHO DDD of 14 g refers to the piperacillin component. IV, intravenous; PO, oral.

**Table 3:** Distribution of the five primary antimicrobial agents according to the documented clinical pathology.

Clinical Pathology	Ceftriaxone	Meropenem	Vancomycin	Piperacillin/Tazobactam	Levofloxacin	Total Prescription Events (*n*)
Pneumonia	35	24	24	19	16	118
Urinary Tract Infection (UTI)	51	7	3	12	14	87
Intra-abdominal Infection	41	19	0	6	0	66
Skin and Soft Tissue Infection	17	13	14	8	3	55
Sepsis Without a Documented Focus	9	3	4	4	0	20
Other Infectious Pathologies	7	6	25	20	35	93
Total Prescriptions per Molecule	160	72	70	69	68	439

Note: Values reflect strict mathematical counts from the institutional database. Rows and columns sum up to individual prescription events, which frequently overlap due to combination therapy regimens in complex patients.

## Data Availability

The data presented in this study are available on request from the corresponding author due to institutional administrative protocol restrictions.

## Abbreviations

The following abbreviations are used in this manuscript:

AMR	Antimicrobial Resistance
ASP	Antimicrobial Stewardship Program
ATC	Anatomical Therapeutic Chemical
AUC	Area Under the Curve
CENETEC	Centro Nacional de Excelencia Tecnológica en Salud
CDI	Clostridioides difficile Infection
COFEPRIS	Comisión Federal para la Protección contra Riesgos Sanitarios
CRE	Carbapenem-Resistant Enterobacteriaceae
DDD	Defined Daily Dose
DUS	Drug Utilization Study
ESBL	Extended-Spectrum Beta-Lactamase
FDA	Food and Drug Administration
GARP	Global Antibiotic Resistance Partnership
HAI	Healthcare-Associated Infection
IMSS	Instituto Mexicano del Seguro Social
LMICs	Low- and Middle-Income Countries
LOS	Length of Stay
MDR	Multidrug-Resistant
MIPD	Model-Informed Precision Dosing
MRSA	Methicillin-Resistant Staphylococcus aureus
PAHO	Pan American Health Organization
PK/PD	Pharmacokinetics/Pharmacodynamics
PROA	Programa de Optimización del Uso de Antimicrobianos
TDM	Therapeutic Drug Monitoring
UTI	Urinary Tract Infection
VISA	Vancomycin-Intermediate Staphylococcus aureus
VRE	Vancomycin-Resistant Enterococci
WHO	World Health Organization
